# L-*myc* Gene Expression in Canine Fetal Fibroblasts Promotes Self-Renewal Capacity but Not Tumor Formation

**DOI:** 10.3390/cells10081980

**Published:** 2021-08-04

**Authors:** So Hee Kim, Bokyung Kim, Jung Hak Kim, Dong-Hoon Kim, Seung Hoon Lee, Dong-Seok Lee, Hong J. Lee

**Affiliations:** 1 College of Medicine and Medical Research Institute, Chungbuk National University, Chungbuk, Cheongju 28644, Korea; kksh4294@naver.com; 2 School of Life Sciences, BK21 FOUR KNU Creative BioResearch Group, Kyungpook National University, Daegu 41566, Korea; mideun@knu.ac.kr; 3 Research Institute, e-biogen Inc., Seoul 04785, Korea; jnhkim1116@gmail.com; 4 Animal Biotechnology Division, National Institute of Animal Science, RDA, Wanju-gun 55365, Jeollabuk-do, Korea; kimdhhj@korea.kr (D.-H.K.); sage@korea.kr (S.H.L.)

**Keywords:** canine fetal fibroblast, induced conditional self-renewing fibroblast cell, somatic cell proliferation, L-*myc*, Inducible promoter

## Abstract

Canines are useful in mammalian preclinical studies because they are larger than rodents and share many diseases with humans. Canine fetal fibroblast cells (CFFs) are an easily accessible source of somatic cells. However, they are easily driven to senescence and become unusable with continuous in vitro culture. Therefore, to overcome these deficiencies, we investigated whether tetracycline-inducible L-*myc* gene expression promotes self-renewal activity and tumorigenicity in the production of induced conditional self-renewing fibroblast cells (iCSFCs). Here, we describe the characterization of a new iCSFC line immortalized by transduction with L-*myc* that displays in vitro self-renewal ability without tumorigenic capacity. We established conditionally inducible self-renewing fibroblast cells by transducing CFF-3 cells with L-*myc* under the tetracycline-inducible gene expression system. In the absence of doxycycline, the cells did not express L-*myc* or undergo self-renewal. The iCSFCs had a fibroblast-like morphology, normal chromosome pattern, and expressed fibroblast-specific genes and markers. However, the iCSFCs did not form tumors in a soft agar colony-forming assay. We observed higher expression of three ES modules (core pluripotency genes, polycomb repressive complex genes (PRC), and MYC-related genes) in the iCSFCs than in the CFF-3 cells; in particular, the core pluripotency genes (OCT4, SOX2, and NANOG) were markedly up-regulated compared with the PRC and MYC module genes. These results demonstrated that, in canine fetal fibroblasts, L-*myc* tetracycline-inducible promoter-driven gene expression induces self-renewal capacity but not tumor formation. This study suggests that L-*myc* gene-induced conditional self-renewing fibroblast cells can be used as an in vitro tool in a variety of biomedical studies related to drug screening.

## 1. Introduction

In 2006, somatic cells were reprogrammed into induced pluripotent stem cells (iPSCs) by 64 viral transduction with 4 transcription factors: *POU5F1* (*Oct3/4*), *SOX2*, *MYC* (C-*myc*), and *KLF4* [1]. In subsequent years, iPSCs were generated from various cell sources, including mouse embryonic fibroblasts, adult mouse tail fibroblasts, and adult human dermal fibroblasts, by retroviral transduction with the 4 key transcription factors [1,2]. These iPSCs show similar characteristics to Embryonic Stem Cells (ESCs) regarding morphology, proliferation, activation of both X chromosomes, pluripotency gene expression and teratoma formation [2,3,4,5]. In an attempt to avoid the ethical and legal issues associated with human tissue biopsies, iPSCs have been generated from fibroblasts of diverse species, such as canine, pig, and rat [6,7,8].In veterinary medicine, canines are used in mammalian preclinical studies because they are larger than rodents and share many diseases with humans. Canine somatic cell-derived iPSCs have been established and characterized [8,9,10]. In animal models, reprogrammed iPSCs show ESC-like characteristics without chromosomal abnormalities [11]. Recently, various biomedical investigations on in vitro drug screening and mammalian preclinical cell therapy studies have characterized different mammalian somatic cell-derived ESC-like iPSCs.

The transcription factor *MYC* is induced during cell proliferation and tumor formation. The most important function of *MYC* is to control cell proliferation, including the regulation of cell–matrix interactions, protein and DNA synthesis, and cell cycle progression [12], which stimulates cyclin–cyclin-dependent kinase complexes [13]. *MYC* also maintains self-renewal and suppresses the expression of differentiation genes to maintain stemness [14]. Moreover, *MYC* enhances reprogramming efficiency and shows consistently high expression in reprogrammed colonies [15]; it inhibits both cell quiescence as well as terminal differentiation [16]. Thus, *MYC* is a multipotent factor that influences many aspects of normal cell behavior related to chromatin remodeling [17]. The *MYC* proto-oncogene family comprises several isoforms, including C-*myc*, *MYCN* (N-*myc*), and *MYCL* (L-*myc*). Some studies have reported that C-*myc* plays a central role in tumorigenicity and self-renewal during iPSC generation and normal embryonic development [1,8,11,18].N-*myc* is similar to C-*myc* in domain structure and is a common factor implicated in human cancer [19]. V-*myc* is the viral homolog of C-*myc* and was first identified in an acute avian retrovirus (MC29) [20]. V-*myc* was previously used to generate the stable human neural stem cell line HB1.F3 and derivative cell lines, which undergo unlimited proliferation and facilitate functional recovery in a mouse stroke model [21]. L-*myc* has a shorter amino acid sequence in the N-terminal domain than C-*myc* [22]. L-*myc* may be a useful factor for promoting self-renewal because it induces less proliferation and tumorigenicity in transformed cells than does C-*myc* [23].

Therefore, we explored in canine fetal fibroblast-3 (CFF-3) cells whether stable, conditionally induced self-renewing fibroblast cells (iCSFCs) could be established with self-renewal activity and low tumorigenicity via transduction with only 1 retroviral vector carrying the tetracycline-inducible promoter-driven L-*myc* gene. Here we present the characterization of a new iCSFCs line immortalized by transduction with tetracycline-inducible promoter-driven L-*myc* that displays self-renewal and non-tumorigenic capabilities in vitro.

## 2. Materials and Methods

### 2.1. Ethics Statement

CFF-3 female begle fetus cell was obtained from the National Institute of Animal Science (RDA). Use of laboratory animals (female begle fetus) for the study was approved by the National Institute of Animal Science (RDA) Institutional Animal Care Committee (IACUC; Certificate 2016-204) and was accordance with the Guide for the care and use of laboratory animals as published by the United States National Institute of Health.

### 2.2. Cell Culture

CFF-3 cells were isolated from a 35-day-old female beagle fetus (Canis lupus familiaris) and cultured in Dulbecco’s modified Eagle’s medium (DMEM, LM 001-05; WelGENE, Daegu, Korea) supplemented with 10% fetal bovine serum (FBS; GenDEPOT, Barker, TX, USA) and 10 μg/mL gentamicin (Gibco, Grand Island, NY, USA). Cultures were maintained at 37 °C in a humidified, 5% CO${}_{2}$ incubator.

### 2.3. Generation of iCSFCs

To induce the proliferation of primary fibroblast cells, we inserted proliferation-inducing genes with a lentiviral vector; transcription of the genes was driven by a tetracycline-inducible promoter, which drove the transcription of the target genes in the presence of the tetracycline derivative doxycycline (Clontech, Palo Alto, CA, USA). The human L-*myc* gene was inserted into the BamHI sites of a lentiviral vector, pDINEO, which contained the tetracycline-driven promoter and a neomycin selective marker. The generated lentivirus vectors were designated pDINEO-L-*myc*.

To generate iCSFCs, the lentivirus carrying pDINEO-L-*myc* was produced in 293FT packaging cells, then used to infect CFF-3 cells. iCSFCs were selected by treatment with G418 (500 μg/mL, Sigma–Aldrich, St. Louis, MO, USA) for 2 weeks. The iCSFCs were maintained and expanded in DMEM with 10% FBS (GenDEPOT), 10 μg/mL gentamicin, and 1 μg/mL doxycycline.

### 2.4. Cytogenetic Analysis

Chromosome identification for somatic cell donors was performed using high-resolution GTG banding. The karyotypes of the iCSFCs (passage 20) were identified using cytogenetic analysis with Giemsa stain (Sigma–Aldrich), and processed via the manufacturer’s protocol.

### 2.5. Reverse Transcription-Polymerase Chain Reaction

We performed reverse transcription-polymerase chain reaction (RT-PCR) with the oligonucleotide primers in Supplementary Table S1. We isolated total RNA from cultured CFF-3 cells and iCSFCs with GeneAll${}^{®}$ RiboEX and a Hybrid-R™ RNA kit (301-001 and 315-150, respectively; GeneAll Biotechnology, Seoul, Korea) with a QIAcube instrument (QIAGEN, Hilden, Germany). We reverse transcribed 1 μg RNA with TOPscript™ RT DryMIX (RT200; Enzynomics, Daejeon, Korea). The cDNA was amplified using POBGEN™ PCR Premix (POSTBIO, Hanam, Korea) over 30 cycles (94 °C for 5 min; then 30 cycles of 10 s at 98 °C, 30 s at 60 °C–65 °C, and 1 min at 72 °C, with a final extension step at 72 °C for 10 min). We amplified glyceraldehyde-3-phosphate dehydrogenase (*GAPDH*) for use as a loading control. Each PCR product was separated by electrophoresis on 2% agarose gels, containing SafeView™ Classic (G108; Applied Biological Materials, Richmond, Canada) at 1 in 10,000 (0.001%), and visualized by a Davinch Chemi Imager (Davinch-K, Seoul, Korea). Each band was densitometrically quantified using ImageJ and normalized to the GAPDH intensity.

### 2.6. Cell Growth and Cell Cycle Analysis

To determine the cell growth rates of iCSFCs, we cultured CFF-3 cells and iCSFCs in the presence or absence of doxycycline. To eliminate the doxycycline effects on proliferation, the cells were cultured in the absence of doxycycline for 7 days. The cell growth rate was determined using the Muse${}^{®}$ Count and Viability Assay Kit (MCH100102; Millipore, Billerica, MA, USA), according to the manufacturer’s instructions, with a Muse${}^{®}$ Cell Analyzer. Briefly, the cells were suspended in phosphate-buffered saline (PBS) and mixed with Muse${}^{®}$ Count and Viability working solution. The cells were analyzed using the Muse${}^{®}$ Cell Analyzer once every 2 days for 2 weeks.

The cell cycle status of the cells was analyzed using the Muse${}^{®}$ Cell Cycle Assay Kit (MCH100106; Millipore) according to the manufacturer’s instructions. The suspended cells were fixed in 70% ice-cold ethanol at −20 °C for 3 h. After they were washed with PBS, the cell pellets were resuspended and incubated in 200 μL Muse${}^{®}$ Cell Cycle reagent. The cell cycle phase distributions were analyzed with the Muse${}^{®}$ Cell Analyzer.

### 2.7. Immunocytochemistry

We performed immunocytochemical determinations of cell type-specific markers in iCSFCs. The cells were grown on ACLAR${}^{®}$ plastic coverslips for 3 days, and fixed in cold 95% ethanol with 5% acetic acid for 10 min. The samples were incubated overnight at 4 °C with primary antibodies: anti-activated leukocyte cell adhesion molecule (ALCAM, sc−74558, Santa Cruz Biotechnology, TX, USA), anti-collagen type I alpha 1 chain (COL1A1, #72026, Cell Signaling, MA, USA), anti-S100 calcium binding protein A4 (S100A4, #13018, Cell Signaling), and anti-heat shock protein 47 (HSP47, NBP1-97491, Novus Biologicals, MO, USA), each at 1:200. Then, the samples were incubated with Alexa Fluor${}^{®}$ 594-conjugated anti-rabbit IgG (1:500) for 1 h at room temperature. Finally, the samples were viewed under a fluorescence microscope (IX71, Olympus, Tokyo, Japan).

### 2.8. Tumor Formation Assay

Tumor formation ability was determined using a soft agar colony-forming assay. We coated each well of a 6-well plate with 2 mL bottom agar mixture (DMEM, 10% FBS, and 0.6% agar). After the bottom layer solidified, we added 2 mL of top agar mixture (DMEM, 10% FBS, and 0.4% agar) containing cells (2.5 × 103/mL) to each well. The cultures were incubated at 37 °C in an incubator with a 5% CO${}_{2}$ atmosphere. Every 5 days, the doxycycline-free growth medium or doxycycline-containing growth medium as layered gently over the cultures. Colony formation was monitored daily using a light microscope. The colonies formed in soft agar were cultured for 14 days, stained with 0.5 mL 0.1% crystal violet (Sigma–Aldrich) for 1 h, then photographed using an Olympus microscope at 40× magnification.

### 2.9. Quantitative RT-PCR

We performed quantitative RT-PCR (qPCR) with the oligonucleotide primers in Supplementary Table S2. The amplifications were carried out in a total volume of 20 μL, containing 10 μL Rotor-Gene SYBR${}^{®}$ Green PCR Kit (204074; QIAGEN), 2 μL of the relevant PCR primers, 7 μL nuclease-free water, and 1 μL DNA template. The thermal cycling conditions included an initial step at 95 °C for 5 min, followed by 40 cycles consisting of 95 °C for 20 s and 60 °C for 30 s. Amplifications were performed using a Rotor-Gene Q system (QIAGEN); we calculated the ΔΔCt data with the Rotor-Gene Q Series software (QIAGEN). All qPCR runs included template-free negative controls. qRT-PCR was performed at triplicate for each replicate and repeated three times for statistical analysis.

### 2.10. Statistical Analysis

All experiments were repeated 3 times. Student’s *t*-test was used to assess statistical significance with thresholds of *p* < 0.05 and *p* < 0.01 for significant and highly significant differences, respectively (CFF-3 versus iCSFCs). We analyzed the data with SigmaPlot version 12 (Systat Software, San Jose, CA, USA).

## 3. Results

### 3.1. Generation of iCSFCs

**Figure 1:**
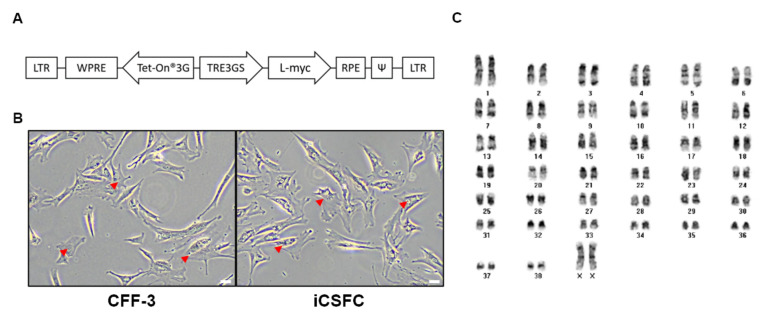
Generation of induced conditional self-renewing fibroblast cell (iCSFC) lines. (**A**) Construction of the lentiviral vector containing L-*myc* (pDINEO–L–*myc*). (**B**) Light microscopy of CFF-3 cells derived from canine fetal fibroblasts at passage 5 and an iCSF cell (iCSFC) line at passage 6 (Scale bars, 50 μm). (**C**) Karyotype analysis of iCSFCs at passage 20 revealed the normal canine karyotype of 78, XX.

A representative clonal iCSFC line was obtained after gene transduction into primitive CFF-3 cells using the pDINEO lentiviral vector encoding L-*myc*, which induced L-*myc* expression in the presence of doxycycline (Figure 1A). After transduction, iCSFCs were selected for 2–3 weeks in G418. The established iCSFC line was stably maintained for more than 20 passages, then characterized. The CFF-3 cells and iCSFCs in the growth medium were observed adherent, fibroblast-like morphologies, with flat, polygonal shapes by an optical microscope. The iCSFCs remained consistent over long-term culture of at least 70 passages, without morphological changes or chromosomal abnormalities (Figure 1B). Upon Giemsa staining and cytogenetic analysis, iCSFCs had a normal karyotype with 78 chromosomes and 2 X sex chromosomes (Figure 1C).

### 3.2. L-*myc* Expression Improved the Proliferation of iCSFCs, Not Promote Tumor Formation

**Figure 2:**
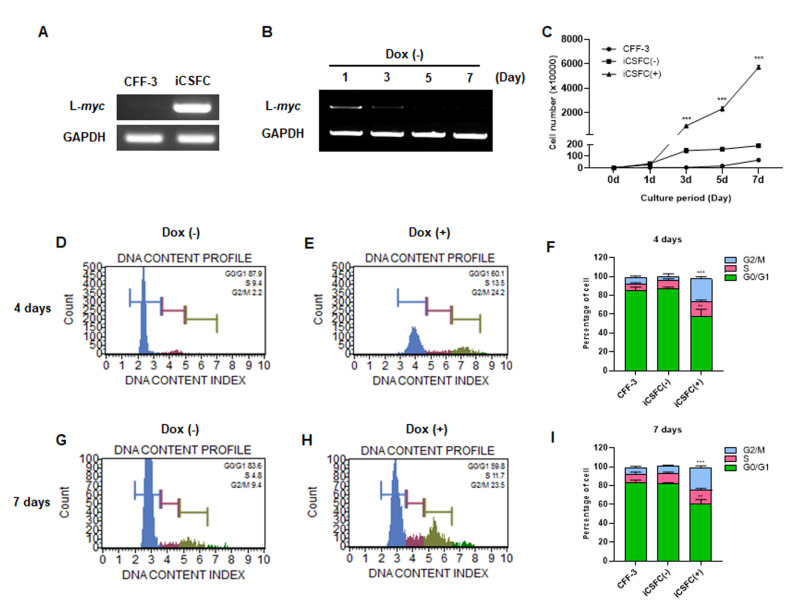
L-*myc* expression in iCSFCs. (**A**) RT-PCR analysis indicate that the L-*myc* gene was over-expressed in iCSFCs in the presence of doxycycline (Dox, 1 μg/mL) following infection with the L-*myc* lentivirus, but not in primary CFF-3 cells. GAPDH is used as the loading control. (**B**) The expression of L-*myc* gradually decreased in the absence of Dox, as measured by RT-PCR. Dox-induced L-*myc* expression stimulated the proliferation of iCSFCs. iCSFCs were cultivated with Dox for 7 days, thereafter without Dox. Dox removal reduced L-*myc* expression and L-*myc*-induced proliferation in iCSFCs as time-dependent manner. (**C**) Growth curves of CFF-3 cells and iCSFCs in the absence (-) and presence (+) of Dox. Cell numbers were counted every 48 h for 5 days. Neither CFF-3 cells nor Dox-starved iCSFCs showed a significant increase in cell number. Expression of L-*myc* in the presence or absence of Dox influenced cell proliferation. (**D**,**E**,**G**,**H**) Cell cycle anlaysis of iCSFCs in the absence and presence of DOX according to the Muse™ cell analyzer. Notably, doxycycline significantly increased the proportion of iCSFCs in G2/M phase. (**F**,**I**) Representative graphs of (**D**,**E**), (**G**,**H**), respectively. Data shown in graphs are the means ± SEM of four independent experiments. **, *p* < 0.01; ***, *p* < 0.001.

To verify the doxycycline-induced L-*myc* expression in iCSFCs, we examined the expression levels of L-*myc* in CFF-3 and iCSFCs in the presence of doxycycline. We found that L-*myc* was expressed in iCSFCs, but not in CFF-3 cells upon doxycycline treatment (Figure 2A). In addition, to confirm the duration of the doxycycline-induced effects in iCSFCs, we examined the L-*myc* expression pattern at various time points after doxycycline removal. RT-PCR showed that the expression of L-*myc* in iCSFCs decreased in a time-dependent manner in the absence of doxycycline (Figure 2B). Subsequently, to investigate whether iCSFCs growth rates increased in a doxycycline-dependent manner, we counted the number of iCSFCs in the absence or presence of doxycycline, along with CFF-3 cells as a control, using the Muse™ Count and Viability Assay Kit at 0, 1, 3, 5, and 7 days. As shown in Figure 2C, treatment with doxycycline caused a marked increase in the proliferation rate of iCSFCs compared with iCSFCs cultured in the absence of doxycycline and CFF-4 cells. Doxycycline treatment of iCSFCs (1 × 10${}^{4}$/mL at day) for 7 days resulted in significantly increased cell numbers by approximately 31-folds (5933 ± 33) compared with untreated iCSFCs (186.33 ± 7.22).

To determine whether the increase in cell numbers was due to proliferation, we analyzed cell cycle progression with a Muse™ Cell Cycle Assay Kit in the presence and absence of doxycycline. The proportion of G2/M phase iCSFCs was low on day 4 (9% ± 4) and day 7 (2% ± 2) in the absence of doxycycline, whereas doxycycline treatment significantly increased this percentage on both day 4 (23% ± 5) and day 7 (24% ± 2) (Figure 2D–I). Complementarily, the proportion of G0/G1 phase cells was higher in the absence of doxycycline.

**Figure 3:**
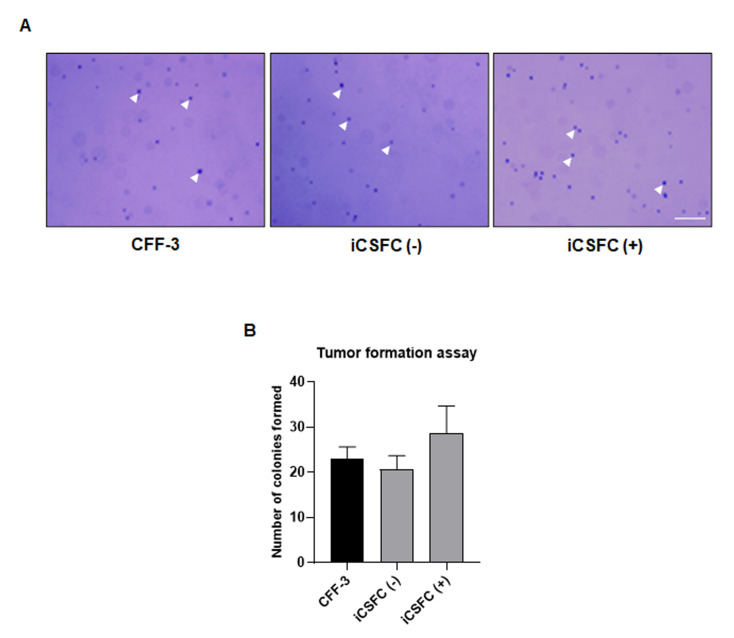
Tumor formation assay in iCSFCs. (**A**) Tumor formation ability of iCSFCs was assessed by colony formation. For tumor formation assay, cells were cultured for 14 days. The iCSFCs did not form colonies on soft agar containing CFF-3 ICSFC (Dox -) or ICSFC (Dox +) (Scale bars, 200 μm). (**B**) The graph represents the number of formed colonies.

To investigate whether iCSFCs formed tumors in vitro, we performed a soft agar colony-forming assay. We observed less growth and fewer iCSFCs in the absence of doxycycline than in the presence of doxycycline (Figure 2C). We were unable to observe tumor formation, and all the cells grew independently. These results indicated that L-*myc* expression in iCSFCs in the presence of doxycycline induced proliferation but did not promote colony formation on soft agar (Figure 3). Thus, these results suggested that, upon doxycycline treatment, L-*myc* expression in iCSFCs conferred the capacity for continuous cell division, resulting in increased cell proliferation. However, L-*myc* expressed iCSFCs did not promote formation.

### 3.3. iCSFCs Maintain Fibroblast Properties In Vitro

**Figure 4:**
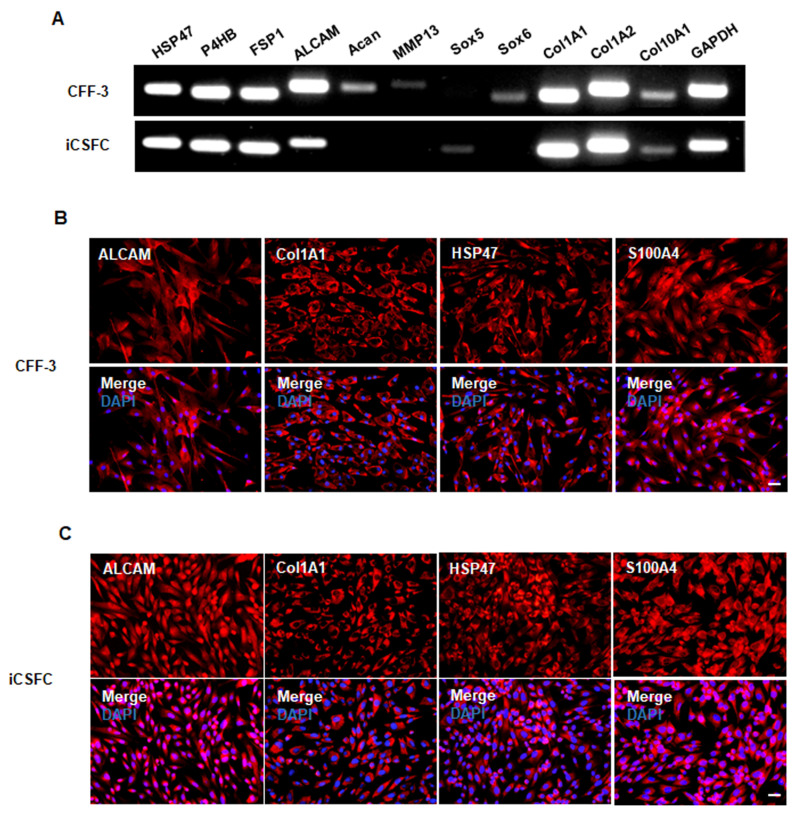
iCSFCs have fibroblast properties. (**A**) Gene expression of fibroblast cell markers was examined by RT-PCR. Most fibroblast genes were expressed in iCSFCs, with the exception of *Acan*, *MMP13* and *Sox6*. *GAPDH* was used as an internal control. (**B**,**C**) Immunocytochemistry analysis of iCSFCs for fibroblast marker proteins. Immunostaining was used to indicate fibroblast markers (red) in CFF-3 cells (**B**) and iCSFCs (**C**), including *ALCAM*, *Col1A1*, *HSP47* and *S100A4*. Cell nuclei were labeled with DAPI (Scale bars, 50 μm).

To analyze the characteristics of the CFF-3 cells and iCSFCs at the genetic level, we performed RT-PCR on well-known fibroblast-specific genes (Supplementary Table S1), including *HSP47*, *P4HB*, *FSP1*, *ALCAM*, *Acan*, *MMP13*, *Sox5*, *Sox6*, *COl1A1*, *COl1A2*, and *COl10A1*. The fibroblast-derived CFF-3 cells expressed the fibroblast markers *HSP47*, *P4HB*, *FSP1*, *ALCAM*, *Acan*, *MMP13*, *Sox6*, *COl1A1*, *COl1A2*, and *COl10A1* but did not express *Sox5* (Figure 4A). The iCSFCs expressed several fibroblast markers, including *HSP47*, *P4HB*, *FSP1*, *ALCAM*, *Sox5*, *COl1A1*, *COl1A2*, and *COl10A1* but lacked detectable levels of *Acan*, *MMP13*, and *Sox6* (Figure 4A). Immunocytochemical staining confirmed the expression of fibroblast marker proteins such as ALCAM, COl1A1, HSP47, and S100A4 by iCSFCs and CFF-3 cells (Figure 4B,C). These results indicated that iCSFCs maintain fibroblast properties in vitro.

### 3.4. Elevated Expression of Three ES Cell Modules (CORE, PRC, and MYC) in iCSFCs

**Figure 5:**
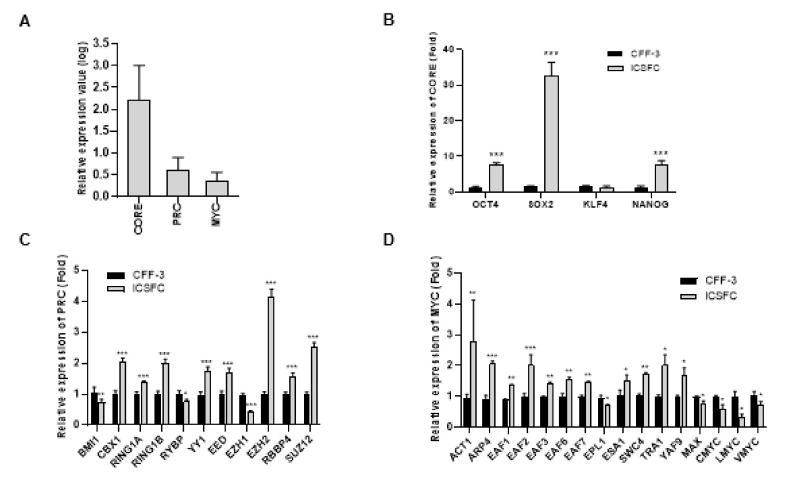
Comparison of the expression of CORE, PRC and MYC module genes in iCSFCs. (**A**) Average gene expression values (log2) of CORE, PRC, and MYC module genes in iCSFCs using values from CFF-3 cells as references; 4 CORE, 11 PRC and 16 MYC module genes were included in the qPCR analysis. (**B**) Expression levels of *OCT4*, *SOX2*, *NANOG* and *KLF4* were compared by qPCR in CFF-3 cells and iCSFCs. In iCSFCs, we observed that 3 CORE module genes (*OCT4*, *SOX2*, *NANOG*) were increased and 1 gene (*KLF4*) was decreased compared to CFF-3 cells. (**C**) Expression comparison of the PRC module genes in iCSFCs and CFF-3 cells. Eight genes showed elevated expression levels in iCSFCs compared with CFF-3 cells. (**D**) Comparison of MYC module gene expression in iCSFCs and CFF-3 cells. Eleven genes showed increased expression levels in iCSFCs compared with CFF-3 cells. Data shown in graphs are the means ±SEM of four independent experiments. *, *p* < 0.05; **, *p* < 0.01; ***, *p* < 0.001.

Fundamental transcriptional subnetworks including core pluripotency factor (CORE), polycomb repressive complex factor (PRC), and *MYC*-related factors (MYC) modules have been shown to participate in preservation of the pluripotency and self-renewability of embryonic stem cells (ESCs) [24]. To determine the molecular signature of the iCSFCs, we used the 3 regulatory gene sets for comparative analyses of gene network activation. The expression levels of the CORE, PRC, and MYC module genes were examined by qPCR analysis (Figure 5A, Supplementary Table S2). The CORE module genes are regulated by specific transcription factors in ESCs, including *POU5F1(OCT4)*, *SOX2*, *NANOG*, and *KLF4* [25]. In iCSFCs, we observed that the expression of 3 genes in the CORE module (*OCT4*, *SOX2*, and *NANOG*) was increased, but not increased expression of *KLF4*, compared to CFF-3 cells (Figure 5B). The PRC module genes are known to be upregulated in fibroblasts and comprise genes inactivated in differentiated cells during development [26]. The iCSFCs showed elevated expression of 8 genes (*CBX1*, *RING1A*, *RING1B*, *YY1*, *EED*, *EZH2*, *RBBP4*, and *SUZ12*) and lower expression of 3 genes (*BMI1*, *RYBP*, and *EZH1*) in the PRC module compared with CFF-3 cells (Figure 5C). iCSFCs showed higher expression of 11 genes (*ACT1*, *ARP4*, *EAF1*, *EAF2*, *EAF3*, *EAF6*, *EAF7*, *ESA1*, *SWC4*, *TRA1*, and *YAF9*) and reduced expression of 5 genes (*EPL1*, *MAX*, C-*myc*, L-*myc*, and V-*myc*) in the MYC module compared to CFF-3 cells (Figure 5D). The core pluripotency genes (*OCT4*, *SOX2*, and *NANOG*) were markedly up-regulated to a much greater extent than PRC and MYC module genes in iCSFCs (Figure 5A). These results suggested that iCSFCs had increased expression of CORE, PRC, and MYC module genes compared with CFF-3 cells. However, we did not observe teratoma formation in a long-term teratoma assay as an in vivo assessment of pluripotency (data not shown).

## 4. Discussion

Canine fetal fibroblast cells (CFFs) are the preferred candidate donor cells for the production of transgenic dogs using somatic cell nuclear transfer, mainly due to their excellent proliferative capacity, developmental ability, and ease of genetic modification [27]. CFFs can be easily manipulated by various techniques such as genetic engineering [27], but they are easily driven to senescence and become unusable with continuous in vitro culture [28,29,30]. In this study, we produced a new conditionally induced self-renewing fibroblast cell (iCSFCs) line immortalized by tetracycline-inducible L-*myc* gene expression that displays in vitro self-renewal ability. These iCSFCs showed a fibroblast-like morphology and expressed fibroblast-specific genes. In addition, three ES modules (core pluripotency genes, polycomb repressive complex genes (PRC), and MYC-related genes) showed elevated expression levels compared with CFFs, as shown in Supplementary Figure S1. However, these cells did not display tumor or teratoma formation. These findings suggest a gene expression profile of iCSFCs more similar to that of stem cells and mouse embryonic fibroblasts than cancer cells. The present study suggests the utility of L-*myc* gene-induced iCSFCs as an in vitro tool in a variety of biomedical studies related to drug screening.

A stable karyotype, which can be analyzed by G-banded karyotyping, is considered a key standard for establishing therapeutically viable ESCs [31]. In human ESCs, genomic abnormalities can accumulate during long-term culture [3,4]. Previously observed genomic abnormalities demonstrated the chromosomal instability of canine induced pluripotent stem cells (ciPSCs) resulting in genomic abnormalities on chromosomes 4, 8, 13, and 16 following prolonged culture [32]. In contrast, the iCSFCs in the present study maintained normal chromosomal patterns after extensive passage (Figure 1B,C). These results suggest that iCSFCs have superior chromosomal stability compared with ciPSCs. One possible explanation is that although induced pluripotent stem cells (iPSCs) and embryonic stem cells (ESCs) share many common features, including similar morphologies and in vitro gene expression profiles, several previous studies have reported a much lower genomic stability in iPSCs than in ESCs [33,34]. The relatively poor genomic stability of iPSCs and their high rate of tumorigenesis in vivo appear to be due, at least in part, to their low fidelity of DNA damage repair. As shown in Figure 3, the iCSFCs in this study did not form tumors in a soft agar colony-forming assay. Therefore, these results indicate that we successfully produced a new iCSFCs line with self-renewal ability and chromosomal stability.

Fibroblast-derived CFF-3 cells and iCSFCs both expressed various fibroblast markers. Thus, it is possible that the new iCSFCs maintain fibroblast properties in vitro (Figure 4). However, as shown in Figure 4A, SOX5 was expressed in the new iCSFCs, a further indication of self-renewal ability, but not in the fibroblast-derived CFF-3 cells. It was reported that both SOX5 and SOX6 are required for optimal proliferation [35]. SOX5 expression is elevated in progenitor cells, whereas SOX5 downregulation in post-mitotic cells is necessary for progression of the differentiation program [36]. These previous studies of SOX5 expression are consistent with our results. SOX6 mediates p53 stabilization and has tumor inhibitory activities in vitro and in vivo [37]. Indeed, SOX6 has been described as a tumor suppressor gene in various cancers [38]. SOX6 directly activates transcription of aggrecan (Acan) [39] and expression of MMP12 in cancer tissue [40]. In the present study, we did not observe detectable levels of SOX6, aggrecan (Acan) or MMP12 in the new iCSFCs (Figure 4A). These results suggest that the new iCSFCs maintain fibroblast properties and do not have tumor activity in vitro.

Recent studies on canine fetal and adult fibroblast cells have described the generation of ciPSCs by retroviral transduction of donor cells with transcription factors in the presence of proliferation stimulators, chemical inhibitors, or DNA methyltransferase inhibitors in order to maintain self-renewal [10,11,32,41,42]. The establishment of iPSCs by retroviral transduction of *MYC* into progenitor cells has proven to be a more practical approach. Several previous studies reported tumor formation by iPSCs overexpressing *MYC in vitro* and in vivo after transduction or transplantation, respectively [18,41]. *MYC* is crucial for promoting self-renewal in somatic cells during the transition to a pluripotent state [16,43], but it also has the potential to induce tumorigenic activity. L-*MYC* promotes cell proliferation but poses a lower oncogenic risk than other MYC family members. L-*MYC* has significantly lower transformation activity in cultured cells compared with other *MYC* members [22,44], and only a small number of human cancers have been associated with the aberrant expression of L-*MYC* [45]. Human stem cells engineered to express L-*MYC* have shown self-renewal and multipotent differentiation capacities in the absence of tumorigenic properties, and human fibroblasts retrovirally transduced with L-*myc* also display normal karyotypes [46]. Thus, similar to the results reported by Nakagawa et al. (2010) [46], we used L-*myc* to generate immortalized iCSFC lines based on its low tumorigenicity. As shown in Figure 2 and Figure 3, we established iCSFCs from donor cells transduced with a single copy of L-*myc* to conditionally induce self-renewal using a tetracycline-inducible gene expression system, which did not promote tumor formation. As a further step, these iCSFC lines immortalized with L-*myc* could represent a safer option for effectively generating canine iPSCs (ciPSCs) with preserved pluripotency and differentiation properties in vitro and in vivo upon the introduction of various transcription factors.

Self-renewal and pluripotency in iPSCs are promoted by the transcription factors *POU5F1*(*Oct4*), *SOX2*, C-*myc*, and *KLF4* [1]; genome-wide regulatory networks have been shown to participate in the preservation of ESC status. A conceptual framework of regulatory networks has recently been developed, which includes protein interactions supporting mouse ESC status, through targeted studies of core pluripotency factors (CORE), polycomb complex factors (PRC), and *MYC*-related factors (*MYC*) [47]. Many genes have been categorized into these 3 transcriptional sub-networks, which are related to specific cellular proliferation patterns [24]. *Oct4*, *Sox2*, and *Nanog* are well-known factors thought to be the master regulators of ES cell pluripotency [2], and *Klf4* is known to induce growth arrest and inhibit cell proliferation by regulating the expression of key cell cycle genes [48,49]. Our results are consistent with those of previous studies of CORE module genes. As shown in Figure 5B, we observed increased expression of 3 CORE module genes (*OCT4*, *SOX2*, *NANOG*) in iCSFCs, but the other CORE module gene (*KLF4*) was decreased compared to CFF-3 cells.

Polycomb group (PcG) proteins are epigenetic repressors essential for cell differentiation and development [50]. A previous study connected PcG protein function with several key processes governing somatic stem cell activity [50]. The two best-characterized PcG complexes, polycomb repressive complex 1 (PRC1) (*CBX1*, *RING1A*, *RING1B*, *BMI1*, *YY1*, *RYBP*, etc.) and PRC2 (*SUZ12*, *EZH2*, *EZH1*, *EED*, *RBBP4*, etc.), are required for maintaining the stemness of embryonic stem cells and many types of adult stem cells. Figure 5C compares the expression of PRC module genes in iCSFCs and CFF-3 cells. The iCSFCs had higher expression of 8 PRC module genes (*CBX1*, *RING1A*, *RING1B*, *YY1*, *EED*, *EZH2*, *RBBP4*, and *SUZ12*) and lower expression of 3 genes (*BMI1*, *RYBP*, and *EZH1*) than CFF-3 cells. The PRC module genes were more highly expressed in iCSFCs than in CFF-3 cells, with EZH2 showing the greatest increase. However, EZH1 was decreased. EZH2 is the functional enzymatic component of PRC2 and is required for healthy embryonic development through epigenetic maintenance of genes responsible for regulating development and differentiation [51]. EZH2 is responsible for the methylation activity of PRC2, and the complex also contains proteins required for optimal functionality of EED and SUZ12 [52]. EZH1 is more abundant in non-proliferative adult organs, while EZH2 expression is tightly associated with proliferation, as evidenced in the aging mouse kidney [53]. Overexpression of Ring1 and YY1 binding protein (RYBP) inhibited anaplastic thyroid cancer cell proliferation and invasion [54]. Therefore, our result showing decreased expression of RYBP and EZH1 with increased EZH2 expression in iCSFCs with self-renewal ability was in concordance with this previous study. *BMI1* (B lymphoma Mo-MLV insertion region 1 homolog) has been reported as an oncogene through its regulation of the cell cycle inhibitors p16 and p19 [55]. In the present study, *BMI1* showed decreased expression. As shown in Figure 3, the iCSFCs did not form tumors in a soft agar, and this is potentially related to their decreased *BMI1* expression. We also observed increased expression of MYC module genes in iCSFCs compared to CFF-3 cells. The MYC module genes stimulate cellular metabolism by activating genes that are controlled by C-*myc* and its related proteins [24]. Our data demonstrate that most MYC module genes are expressed in the iCSFCs. However, as shown in Figure 5D, C-*myc*, L-*myc*, and V-*myc* were decreased. The C-myc protein can act as a modulator of its own gene transcription, and our results are consistent with the hypothesis that a negative feedback mechanism contributes to genetic the regulation of C-myc in normal cells [56]. Thus, the decreased C, L, and V-*myc* in L-*myc*-expressing iCSFCs may be the result of a negative feedback mechanism. Similar to the results presented in supplementary Figure S1, previous studies [24,57] reported that CORE module gene levels and MYC module gene levels are increased in ESCs, whereas PRC module genes are expressed at low levels. Mouse embryonic fibroblasts express low levels of CORE and MYC module genes but high levels of the PRC module genes. Cancer cells have low expression of CORE and PRC module genes but high expression of MYC module genes [24]. We found that the expression of CORE module genes (*OCT4*, *SOX2*, and *NANOG*) in iCSFCs was markedly up-regulated compared with PRC and MYC module genes, indicating that CORE module genes were increased by somatic reprogramming of iCSFCs. CORE module genes are key developmental homeodomain proteins that are suppressed in differentiated cells; self-renewal is sustained by a combinatorial network involving these genes [58]. Therefore, these results indicated that the iCSFCs had an expression pattern more similar to ESCs than fibroblasts and cancer cells.

Dogs have great potential as an animal model for human genetic diseases compared to the more widely accepted rodent models used in preclinical research [59,60]. Canines have been used to support preclinical studies because they are larger and more accurately reflect human responses to diseases than rodents. The risks associated with the application of new therapies may be better represented in canine [57,61,62] than in mouse [63,64] models, and safety and efficacy studies in dogs should therefore be considered before conducting clinical trials in humans [65]. Canines share many physiologic features with humans, as well as various hereditary diseases, such as van den Ende-Gupta syndrome, Raine syndrome, neurodegenerative disorders and inherited musculoskeletal diseases [66,67,68]. Thus, canine models may support the development of new therapeutic approaches for human diseases, and iCSFCs may be used for human disease models in the near future.

## 5. Conclusions

The use of an L-*myc* transduction system for iPSCs may be beneficial for future clinical applications and reprogramming research. Moreover, iCSFCs can be used in a variety of biomedical research studies related to cell therapy and drug screening. These cells can also be used as the basis for establishing alternative cellular models for several animal- or human-specific diseases.

## Data Availability

The datasets and materials used and/or analyzed during the current study are available from the corresponding author on reasonable request.

## Supplementary Materials

The following are available at https://www.mdpi.com/article/10.3390/cells10081980/s1, Figure S1: The three sets of genes that are activated or repressed in iCSFCs, Table S1: PCR Primer Sequences for Cell Type-Fibroblast Markers (All Canis lupus familiaris), Table S2: qPCR Primer Sequences for CORE, PRC, MYC module Markers (All Canis lupus familiaris).

## Sample Availability

Samples of the compounds are available from the authors.

## Abbreviations

The following abbreviations are used in this manuscript:

CFF-3	Canine fetal fibroblast-3
iCSFCs	induced conditional self-renewing fibroblast cells
ESCs	embryonic stem cell
iPSCs	induced pluripotent stem cells
RT-PCR	reverse transcription-polymerase chain reaction
Dox	doxycycline
CORE	core pluripotency factors
PRC	polycomb repressive complex factor
MYC	MYC-related factors
qPCR	quantitative RT-PCR
ciPSCs	canine induced pluripotent stem cells
DMEM	Dulbecco’s modified Eagle’s medium
FBS	fetal bovine serum
GAPDH	glyceraldehyde-3-phosphate dehydrogenase
PBS	phosphate-buffered saline
ALCAM	activated leukocyte cell adhesion molecule
COL1A1	collagen type I alpha 1 chain
S100A4	S100 calcium binding protein A4
HSP47	heat shock protein 47
MEF	mouse embryonic fibroblast
