# Supplementary material for: L-myc Gene Expression in Canine Fetal Fibroblasts Promotes Self-Renewal Capacity but Not Tumor Formation
Source: Cells. 2021 Aug 4;10(8):1980. doi: 10.3390/cells10081980 (PMC8391401; doi:10.3390/cells10081980)
Supplement: Supplementary file 1 [file cells-10-01980-s001.zip › Supplementary Table S1.pptx]

## Slide 1
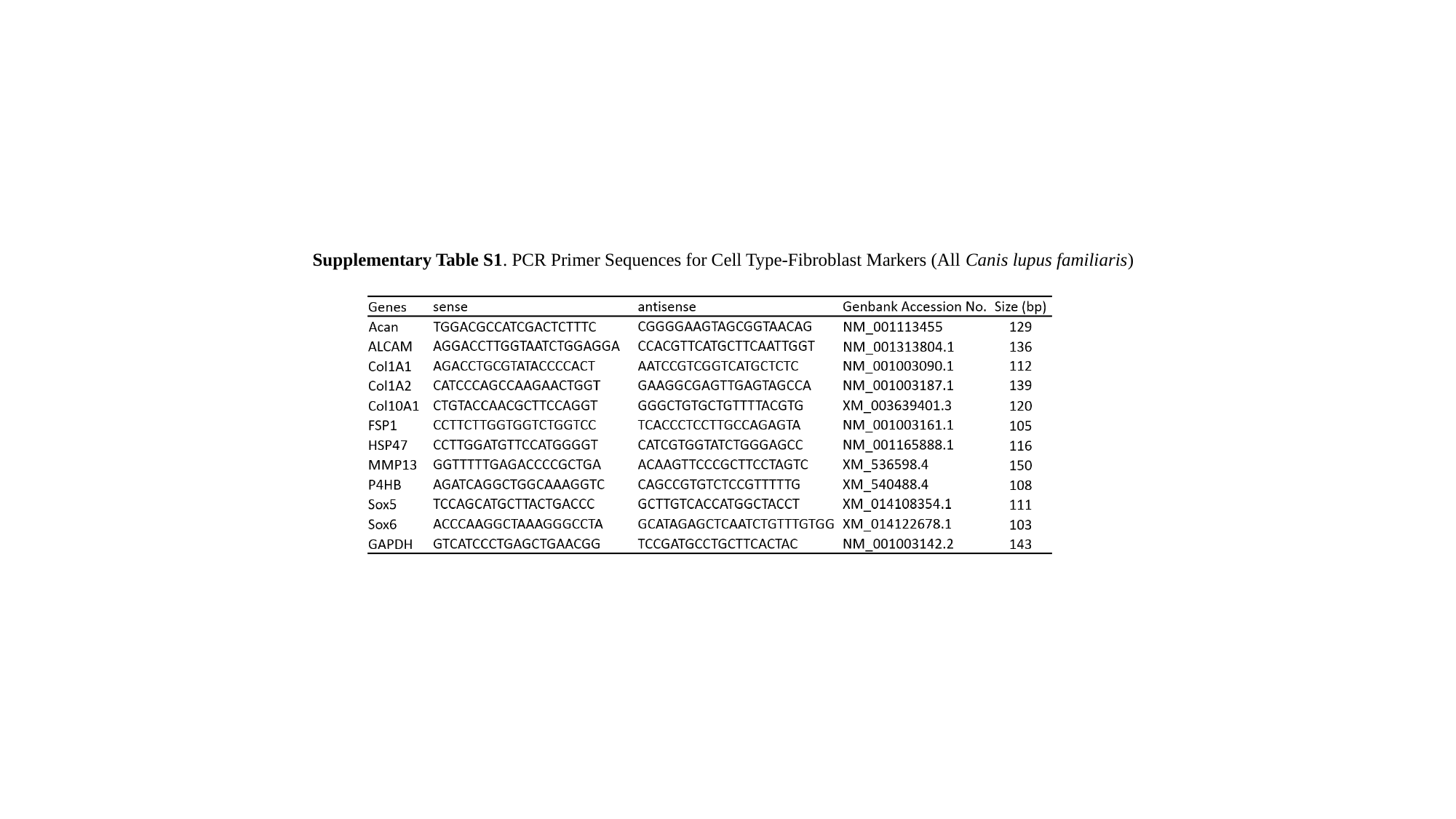

Supplementary Table S1. PCR Primer Sequences for Cell Type-Fibroblast Markers (All Canis lupus familiaris)
